# OsWRKY76 positively regulates drought stress *via* OsbHLH148-mediated jasmonate signaling in rice

**DOI:** 10.3389/fpls.2023.1168723

**Published:** 2023-04-05

**Authors:** Mingxing Zhang, Ranran Zhao, Kai Huang, Zhiqi Wei, Boya Guo, Shuangzhan Huang, Zhao Li, Wenzhu Jiang, Tao Wu, Xinglin Du

**Affiliations:** Jilin Province Engineering Laboratory of Plant Genetic Improvement, College of Plant Science, Jilin University, Changchun, China

**Keywords:** drought stress, jasmonate signaling, OsWRKY76, OsbHLH148, rice (*Oryza sativa*)

## Abstract

Drought stress is a major environmental threat that limits plant growth and crop productivity. Therefore, it is necessary to uncover the molecular mechanisms behind drought tolerance in crops. Here, OsWRKY76 positively regulated drought stress in rice. *OsWRKY76* expression was induced by PEG treatment, dehydration stress, and exogenous MeJA rather than by no treatment. Notably, *OsWRKY76* knockout weakened drought tolerance at the seedling stage and decreased MeJA sensitivity. *OsJAZ12* was significantly induced by drought stress, and its expression was significantly higher in *OsWRKY76*-knockout mutants than in wild-type ZH11 under drought stress. Yeast two-hybrid and bimolecular fluorescence complementation assays showed that OsWRKY76 interacted with OsJAZ12. OsWRKY76 weakened the interaction between OsbHLH148 and OsJAZ12 in yeast cells. The OsJAZ12 protein repressed the transactivation activity of OsbHLH148, and this repression was partly restored by OsWRKY76 in rice protoplasts. Moreover, *OsDREB1E* expression was lower in *OsWRKY76*-knockout mutants than in wild-type ZH11 under drought stress, but it was upregulated under normal growth conditions. Yeast one-hybrid, electrophoretic mobility shift, and dual-luciferase assays showed that OsWRKY76 and OsbHLH148 bound directly to the *OsDREB1E* promoter and activated *OsDREB1E* expression in response to drought stress. These results suggest that OsWRKY76 confers drought tolerance through OsbHLH148-mediated jasmonate signaling in rice, offering a new clue to uncover the mechanisms behind drought tolerance.

## Introduction

Drought is one of the most serious environmental stressors in crops (Sinclair, 2011). Rice (*Oryza sativa*) is a staple crop that feeds over half of the world’s population (Seck et al., 2012). Since the rice cultivation process relies heavily on fresh water resources, shortages of fresh water availability and adverse climate change threaten rice growth and productivity (Hu and Xiong, 2014). This necessitates the identification of drought-resistance genes to create drought-tolerant rice varieties.

Plants have developed complex strategies against drought stress, such as modifying root growth, regulating osmotic substances to maintain intracellular water content, and scavenging excessive reactive oxygen species (ROS) to prevent ROS toxicity caused by drought stress (Rodrigues et al., 2019; Gupta et al., 2020). Numerous rice genes have been reported to play vital roles in drought stress. HDG11 confers drought tolerance and increases grain yield in rice by improving levels of soluble sugar and proline, and ROS-scavenging enzyme activities (Yu et al., 2013). OsTF1L enhances stomatal closure and lignin biosynthesis and positively regulates drought tolerance in rice (Bang et al., 2019). *AtNOG1*-*1* or *AtNOG1*-*2* overexpression in rice increases drought tolerance without affecting grain yield, whereas *OsNOG1*-*RNAi* rice plants are more sensitive to drought stress than wild-type plants (Pant et al., 2022). Moreover, the primary signal caused by drought is a water deficit, leading to osmotic stress. Under osmotic stress created by PEG treatment, the shoots and roots of *OsWIH2*-overexpressing rice plants are longer than those of wild-type rice plants (Gu et al., 2021). *OsWIH2*-overexpressing rice plants have a significantly higher survival rate and a lower water loss rate compared with wild-type rice plants (Gu et al., 2021). Glutathione peroxidase 1 (GPX1) acts as a redox transducer and confers osmotic stress tolerance in rice by interacting with bZIP68 and promoting the oxidation of bZIP68 (Zhou et al., 2022).

Phytohormones, such as jasmonates (JAs), play a crucial role in responding to stress (Y. Wang et al., 2021). JAs are a general term for JA and its active derivatives, which comprise its isoleucine conjugate (JA-Ile) and its methyl ester (MeJA). JAs participate in plant growth and development, such as stomatal opening, root formation, and leaf senescence (H. Huang et al., 2017). Furthermore, a number of recent reports have revealed that JAs can increase plant stress tolerance through the JA signaling pathway. The core component of the JA signaling pathway is JA-Ile (Marquis et al., 2020). Under normal growth conditions, intracellular JA-Ile levels are very low, and JA signaling occurs during the inhibition state. JAZ proteins that feature the jasmonate zinc-finger inflorescence meristem domain interact with various transcription factors (TFs) participating in the JA signaling pathway. JAZ proteins can suppress this TF activity by recruiting the co-repressor topless (TPL) and the adaptor protein of JAZ (NINJA) to form a JAZ–NINJA–TPL complex or histone-modifying proteins HDA6 and HDA19 (Pauwels et al., 2010; Zhu et al., 2011; Ke et al., 2015). When plants suffer from stress, JA-Ile levels are elevated in the cytosol, and JA-Ile is transported into the nucleus and promotes the interaction of JAZ proteins with the F-box protein COI1 within a kinetochore protein 1–cullin 1–F-box protein COI1 (SCF^COI1^) complex, which leads to JAZ protein degradation *via* the 26S proteasome and activation of various JA- and stress-responsive genes (Mosblech et al., 2011; Wasternack and Hause, 2013; Wasternack and Song, 2017).

Basic helix–loop–helix (bHLH) TFs play a vital role in the JA signaling pathway. In *Arabidopsis thaliana*, the expression of the bHLH family *MYC2* is rapidly upregulated by JA (Lorenzo et al., 2004). MYC2 and its paralogs MYC3 and MYC4 interact with JAZ proteins, are directly repressed by JAZ proteins, and positively regulate JA-dependent plant defense (Fernández-Calvo et al., 2011; Schweizer et al., 2013). *OsbHLH148*, whose expression is rapidly increased under dehydration stress and MeJA treatment, confers drought tolerance in rice and regulates the JA signaling pathway (Seo et al., 2011). OsbHLH148 interacts with some OsJAZ proteins, including OsJAZ7, OsJAZ8, OsJAZ11, and OsJAZ12, but OsJAZ12 interacts with OsCOI1 only in the presence of coronatine, causing OsJAZ12 degradation (Seo et al., 2011). In addition, OsJAZ1 attenuates drought tolerance in rice and negatively regulates the JA signaling pathway (Fu et al., 2017). OsbHLH148–OsJAZ model could act as a key regulator in drought stress and jasmonate signaling.

Studies have identified that some WRKY TFs participate in drought stress in rice. OsWRKY47 positively regulates tolerance to water deficit stress (Raineri et al., 2015). OsWRKY11 activates the transcription of the drought-responsive gene *RAB21* and enhances drought stress tolerance (Lee et al., 2018). However, OsWRKY55 positively regulates *OsAP2-39* expression and negatively regulates drought stress (K. Huang et al., 2021). OsWRKY5 represses ABA-induced stomatal closure and weakens drought tolerance in rice (Lim et al., 2022). Furthermore, WRKY proteins interact with other proteins, such as kinases and TFs, regulate their downstream target genes, and then form complex regulatory networks. OsMPK3, OsMPK4, and OsMPK7 interact with OsWRKY30 and phosphorylate OsWRKY30, which is essential for OsWRKY30 to confer drought tolerance in rice (Shen et al., 2012). OsWRKY87 interacts with SAPK10 and is phosphorylated by it. OsWRKY87 can activate *ABF1* transcription and enhance rice drought tolerance (Yan et al., 2021). However, the underlying molecular mechanisms by which other OsWRKY TFs participate in drought stress have not been fully understood.

Our previous data showed that OsWRKY76 interacts with OsbHLH148 and coordinately plays a positive role in cold tolerance in rice (Zhang et al., 2022). Moreover, OsbHLH148 confers drought tolerance in rice and regulates the JA signaling pathway (Seo et al., 2011). However, whether and how OsWRKY76 mediates the drought stress response and the JA signaling pathway in rice is unknown. In this study, we proposed that OsWRKY76 plays a crucial role in regulating drought stress and jasmonate signaling in rice. *OsWRKY76* knockout weakened drought tolerance at the seedling stage and decreased MeJA sensitivity. OsWRKY76 interacted with OsJAZ proteins and activated OsbHLH148 transcriptional activity by interfering with the association of OsJAZ12 with OsbHLH148. OsWRKY76 and OsbHLH148 directly activated the transcription of *OsDREB1E* under drought stress.

## Materials and methods

### Plant materials and growth conditions

The *OsWRKY76*-knockout mutants and *OsbHLH148*-knockout mutants in the background of rice variety ZH11 (*Oryza sativa* L. ssp. *japonica*) were obtained from a mutant library (BIOGLE, Changzhou, Jiangsu, China) using a CRISPR/Cas9 genome editing approach. All homozygous T_3_ seedlings (*w76*-*1*, *w76*-*2*, *b148*-*1*, and *b148*-*2*) were used for this study. All rice seedlings were planted in a growth room (80% relative humidity and PPFD 75 µmol/m^2^s) with a cycle of 14 h light at 30°C and 10 h dark at 26°C.

### Drought-tolerance assays and treatments

For analysis of drought stress tolerance, 2-week-old seedlings of wild-type ZH11 rice and *OsWRKY76*-knockout mutants (*w76*-*1* and *w76*-*2*) were transferred to the hydroponic culture medium with or without 20% PEG 6000 for 10 days, and their survival rates were counted after recovery for 10 days. To further test the effect of OsWRKY76 on drought tolerance, 10-day-old seedlings of wild-type ZH11 rice and *OsWRKY76*-knockout mutants (*w76*-*1* and *w76*-*2*) grown in soil suffered from drought stress for one week by stopping irrigation until the leaves of wild-type ZH11 were completely wilted. After recovery with rewatering for one week, the survival rates of the plants were counted visually.

For drought stress and MeJA treatments, two-week-old ZH11 seedlings were treated under dehydration stress (air-dried), 20% PEG 6000 treatment, and MeJA (100 μM) treatment (Fu et al., 2017). The leaves of the ZH11 seedlings were harvested at different time points after treatment.

For the analysis of MeJA on seed germination, seeds of wild-type ZH11 rice and *OsWRKY76*-knockout mutants (*w76*-*1* and *w76*-*2*) were germinated on 1/2 MS medium containing 0, 5, or 50 μM MeJA for 3 days, and then germination was scored. To determine the effect of MeJA on seedling development, 1-d-old seedlings of wild-type ZH11 rice and *OsWRKY76*-knockout mutants (*w76*-*1* and *w76*-*2*) were transferred to 1/2 MS medium containing 0, 5, or 50 μM MeJA for 5 days, and then shoot length and root length were measured.

### Reverse transcription quantitative PCR analysis

Total RNA was extracted from rice leaves using TRIzol reagent (Tiangen, Beijing, China), and then reverse transcription was performed using a StarScript II RT Kit with gDNA Remover (Genstar, Beijing, China). Reverse transcription quantitative PCR (RT-qPCR) was performed with the SYBR Green-based Fast Mixture (Genstar, Beijing, China) using an Mx3005P instrument (Stratagene, La Jolla, CA, USA). Relative expression levels of genes were calculated by normalization to *OsActin* and *Ubiquitin5* genes (Mao et al., 2019).

### 3, 3’-diaminobenzidine staining

To determine the presence of hydrogen peroxide (H_2_O_2_) in the rice leaves, 3, 3’-diaminobenzidine (DAB) staining (Wang et al., 2021) was performed using two-week-old rice seedlings of wild-type ZH11 rice and *OsWRKY76*-knockout mutants (*w76*-*1* and *w76*-*2*) with or without dehydration treatment. The rice leaves were cut into 2-cm pieces, immersed in 1 mg ml^–1^ DAB in 50 mM Tris–acetate buffer (pH 5.0), and then vacuum infiltrated for 40 min. After incubation at 30°C for 3 h in the dark, the rice leaves were immersed in 95% ethanol until the chlorophyll was completely removed.

### Yeast two- and three-hybrid assays

For the yeast two-hybrid assay, according to the Yeast Protocol Handbook of Clontech, the full-length coding sequences (CDSs) of *OsbHLH148*, *OsJAZ7*, *OsJAZ8*, *OsJAZ11*, and *OsJAZ12* were cloned and fused into the vector pGADT7 (AD) to generate AD-OsbHLH148, AD-OsJAZ7, AD-OsJAZ8, AD-OsJAZ11, and AD-OsJAZ12, respectively, as prey. The full-length CDSs of *OsWRKY76* and *OsbHLH148* were inserted into the vector pbridge (BD) to form BD-OsWRKY76 and BD-OsbHLH148, respectively, as bait. In the yeast three-hybrid assay, according to the Yeast Protocol Handbook of Clontech, the full-length CDSs of *OsWRKY76* were inserted into the BD-OsbHLH148 vector to form BD-OsbHLH148-OsWRKY76 in which *OsWRKY76* driven by the Met25 promoter was expressed in the absence of methionine. These prey and bait vectors were transformed into Y2HGold cells grown on SD/-Trp-Leu dropout plates for the yeast two-hybrid assay and into Y190 cells grown on SD/-Trp-Leu-Met dropout plates for the yeast three-hybrid assay. The positive transformants were grown on SD/-Trp-Leu and SD/-Trp-Leu-His dropout plates for the yeast two-hybrid assay, and β-galactosidase activity levels were detected for the yeast three-hybrid assay.

### Bimolecular fluorescence complementation

To confirm the *in vivo* interaction between OsWRKY76 and OsJAZ12, a bimolecular fluorescence complementation (BiFC) assay was performed. The CDSs of *OsWRKY76* and *OsJAZ12* were amplified and fused into the pxy103 (nYFP) and apXY105 (cYFP) vectors, respectively. Thus, *OsWRKY76-nYFP* and *cYFP-OsJAZ12* fusion constructs were obtained. cYFP or cYFP-OsJAZ12 and nYFP or OsWRKY76-nYFP were transformed into *Arabidopsis* protoplasts (S. C. Wang et al., 2005). Fluorescence signals were analyzed using a Zeiss Axio Observer A1 (Carl Zeiss, Jena, Germany).

### Gal4-dependent chimeric transactivation assay

To determine OsbHLH148 transcriptional activity, the full-length CDS of *OsbHLH148* was amplified and fused into the Gal4 DNA-binding domain (GD), creating the GD-b148 effector vector. Moreover, the full-length CDSs of OsWRKY76 and OsJAZ12 were cloned into the effector vector without GD. Combinations of these effector vectors, reporter vector (*35S*-*Gal4:FLUC*) (firefly luciferase, FLUC), and internal control (*35S:RLUC*) (Renilla luciferase, RLUC) were transformed into rice protoplasts followed by incubation at 30°C for 15 h in the dark to allow transient expression. FLUC and RLUC activities were measured using a Dual-Luciferase reporter assay system (Promega, Madison, Wisconsin, USA).

### Yeast one-hybrid assay

The yeast one-hybrid assay was performed as previously described (Lin et al., 2007). An upstream 2000-bp sequence of *OsDREB1E*’s start codon as a promoter sequence of *OsDREB1E* was cloned and fused into the pLacZi2μ vector to generate *OsDREB1Epro : LacZ*. The full-length CDSs of *OsWRKY76* and *OsbHLH148* were ligated into the pJG4-5 (GAD) vector to form GAD-OsWRKY76 (GAD-W76) and GAD-OsbHLH148 (GAD-b148), respectively. The GAD, GAD-W76, and GAD-b148 constructs were separately co-transformed with the *OsDREB1Epro : LacZ* vector into the yeast strain EGY48. The positive transformants were identified using SD/-Trp-Ura dropout plates containing X-Gluc for blue color development.

### Electrophoretic mobility shift assay

Electrophoretic mobility shift assay (EMSA) was conducted as previously described (Gao et al., 2019). The full-length CDS of OsWRKY76 was inserted into the pGEX-4T-3 vector to fuse it to the glutathione S-transferase (GST) coding region and to create a recombinant vector encoding a GST-WRKY76 fusion protein. The recombinant vector and empty GST vector were then transformed into the *E*. *coli* strain BL21 (DE3), which was purified using GST Resin. The 6-FAM 5’ end-labeled and unlabeled *OsDREB1E* probe was synthesized by a Bio-Tech Company (Sangon, Shanghai, China). Fluorescence signals in the gel were detected using a Tanon 5200 Multi imaging system (Tanon, Shanghai, China).

### Dual-luciferase assay

The *OsDREB1E* promoter was cloned into the double vector pGreenII 0800-LUC, in which the *OsDREB1E* promoter was fused with the *FLUC* reporter gene, and the *Renilla luciferase* gene was driven by the 35S promoter as an internal control. The full-length CDSs of *OsWRKY76* and *OsbHLH148* were inserted into pCAMBIA1300 as the effector vector. The effector vectors were co-transformed with the double vector into rice protoplasts, followed by incubation at 30°C for 15 h in the dark to allow transient expression. The transformed protoplasts were treated with and without 10% PEG 6000 treatment for 20 min. The FLUC and RLUC activities were measured with a Dual-Luciferase reporter assay system (Promega, Madison, Wisconsin, USA).

### Statistical analysis

Based on no fewer than three independent experiments (n ≥ 3), results are presented as the mean ± SD. Mean differences between two groups were compared using Student’s *t*-test and between three or more groups using one-way ANOVA. For Student’s *t*-test, asterisks represent the *P*-values (*, *P* < 0.05; **, *P* < 0.01). For ANOVA, different letters represent significant mean differences (*P* < 0.05) measured using Duncan’s multiple range test (DMRT).

### Primers and accession numbers

The sequences of all primers used for this study are listed in **Table S1**. The sequence data from this study can be found in the Rice Genome Annotation Project Database (http://rice.uga.edu./), and the accession numbers of genes are listed in **Table S2**.

## Results

### Expression of the *OsWRKY76* gene in response to drought stress and MeJA treatment

**Figure 1:**
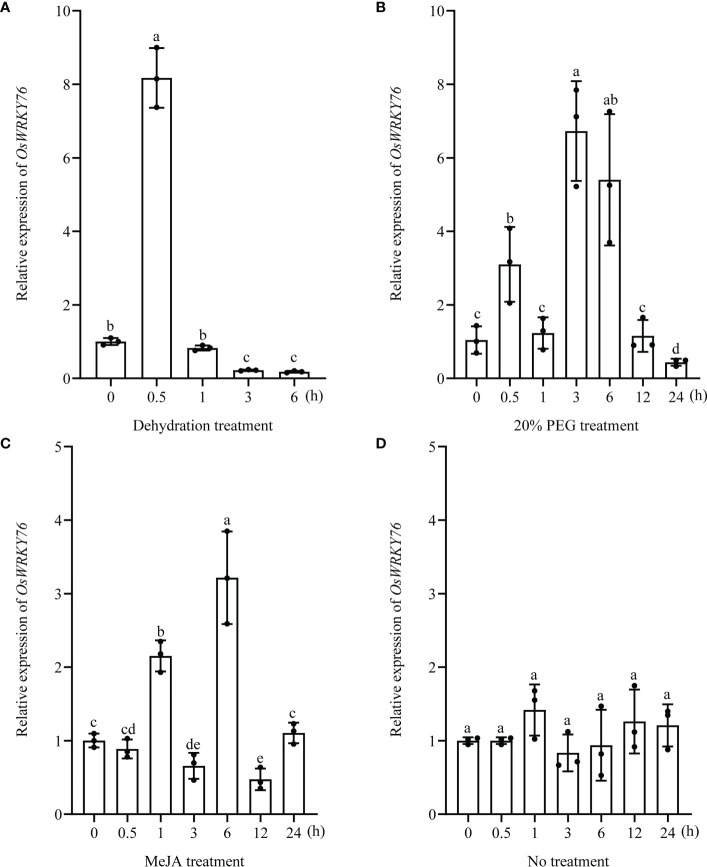
*OsWRKY76* responds to drought stress and MeJA treatment. **(A–D)** Reverse transcription quantitative PCR (RT-qPCR) analysis showing the expression levels of *OsWRKY76* under dehydration stress **(A)**, 20% PEG treatment **(B)**, 100 μM exogenous MeJA treatment **(C)**, and no treatment **(D)** in two-week-old seedlings of wild-type rice (n = 3; *P* < 0.05; one-way ANOVA).

To investigate the responses of *OsWRKY76* to drought stress and MeJA treatment, we examined the expression of *OsWRKY76* under PEG treatment, dehydration stress, and exogenous MeJA in two-week-old seedlings of wild-type rice using reverse RT-qPCR analysis. *OsWRKY76* expression was induced rapidly within 30 min of exposure to dehydration stress, showing an 8-fold increase over that in control plants and then showing a gradual descent over a period of 6 h (**Figure 1A**). *OsWRKY76* was strongly upregulated by PEG treatment and exogenous MeJA (**Figures 1B, C**). By contrast, *OsWRKY76* expression showed no significant differences under no treatment (**Figure 1D**). These results suggest that *OsWRKY76* can respond to drought stress and MeJA treatment and may play an important role in the drought tolerance of rice and the JA signaling pathway.

### OsWRKY76 confers drought tolerance in rice

**Figure 2:**
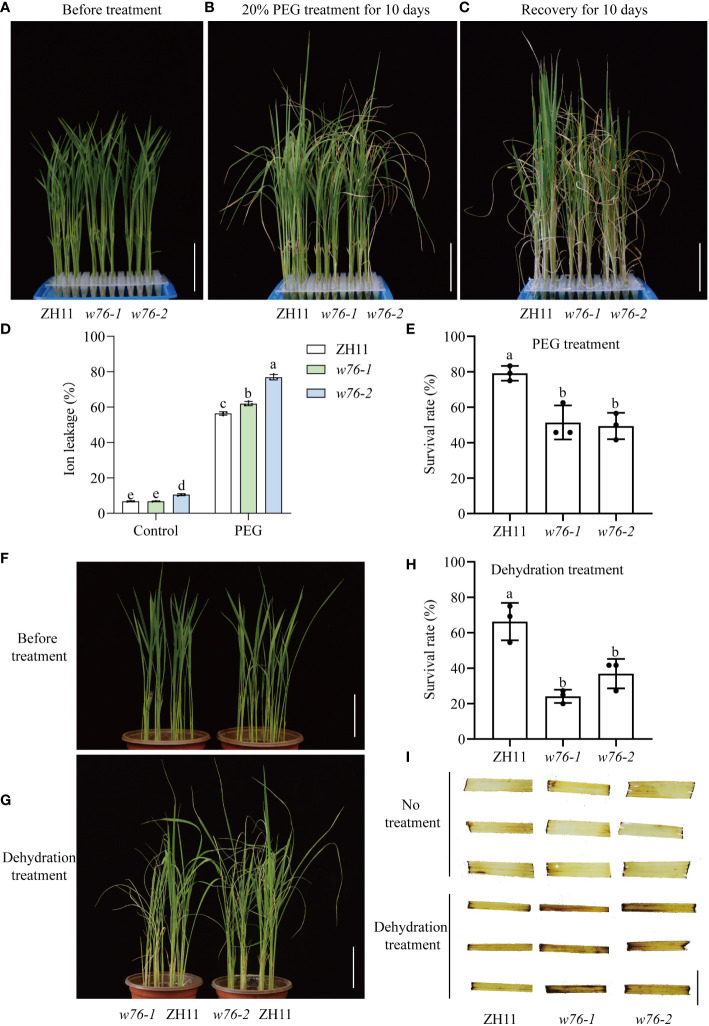
OsWRKY76 confers drought tolerance in rice. **(A–C)** Phenotypes of wild-type ZH11 and *OsWRKY76*-knockout mutants (*w76*-*1* and *w76*-*2*) before treatment **(A)**, with 20% PEG treatment for 10 days **(B)**, and after recovery for 10 days **(C)**. Scale bars, 5 cm. **(D)** Ion leakage rate in leaf cells of ZH11 and *OsWRKY76*-knockout mutants (*w76*-*1* and *w76*-*2*) under no treatment and 10 days of 20% PEG treatment. **(E)** Survival rates of ZH11 and *OsWRKY76*-knockout mutants (*w76*-*1* and *w76*-*2*) after 10 days of recovery. **(F, G)** Phenotypes of wild-type ZH11 and *OsWRKY76*-knockout mutants (*w76*-*1* and *w76*-*2*) under normal conditions **(F)** and after 7 days of rewatering **(G)**. Scale bars, 5 cm. **(H)** Survival rates of ZH11 and *OsWRKY76*-knockout mutants (*w76*-*1* and *w76*-*2*) after 7 days of rewatering. **(I)** 3, 3’-diaminobenzidine (DAB) staining in wild-type ZH11 and *OsWRKY76*-knockout mutants (*w76*-*1* and *w76*-*2*) under normal conditions and 6-h dehydration treatment. Scale bars, 1 cm. (n = 3; *P* < 0.05; one-way ANOVA).

To test the effect of OsWRKY76 on the drought tolerance of rice, *OsWRKY76*-knockout mutants (*w76*-*1* and *w76*-*2*) were obtained using a CRISPR/Cas9 genome editing approach in the *japonica* rice accession ‘ZH11’ background, resulting in frameshift mutations (Zhang et al., 2022). Analysis of drought stress tolerance showed no significant differences in two-week-old rice seedling growth between wild-type ZH11 and *OsWRKY76*-knockout mutants (*w76*-*1* and *w76*-*2*) under normal conditions (**Figures 2A, F**). By contrast, *w76*-*1* and *w76*-*2* plants treated with 20% PEG showed more severe wilting, chlorosis, electrolyte leakage, and water loss than wild-type ZH11 (**Figures 2B–D** and **S1**), together with lower survival rates after 10 days of recovery (**Figure 2E**). We further investigated drought stress conditions. When watering was stopped for 7 days and then resumed for 7 days, *OsWRKY76*-knockout mutants (*w76*-*1* and *w76*-*2*) showed more severe wilting and lower survival rates than wild-type ZH11 (**Figures 2G, H**). These observations suggest that loss of OsWRKY76 function renders rice more sensitive to drought stress than the wild type.

Drought stress triggers ROS accumulation in rice cells, and ROS are biomarkers of rice drought tolerance (Li et al., 2021). Using DAB staining, we determined the presence of H_2_O_2_ in rice leaves. DAB staining was light in wild-type ZH11 and *OsWRKY76*-knockout mutants (*w76*-*1* and *w76*-*2*) under normal conditions (**Figure 2I**). However, DAB staining was stronger in *OsWRKY76*-knockout mutants (*w76*-*1* and *w76*-*2*) than in wild-type ZH11 after 6 h of dehydration treatment (**Figure 2I**). These leaf phenotypes demonstrated that more H_2_O_2_ accumulated in *OsWRKY76*-knockout mutants (*w76*-*1* and *w76*-*2*) than in wild-type ZH11 after drought stress. Taken together, these results suggest that OsWRKY76 positively regulates drought tolerance by repressing ROS accumulation.

### OsWRKY76 positively regulates JA signaling

**Figure 3:**
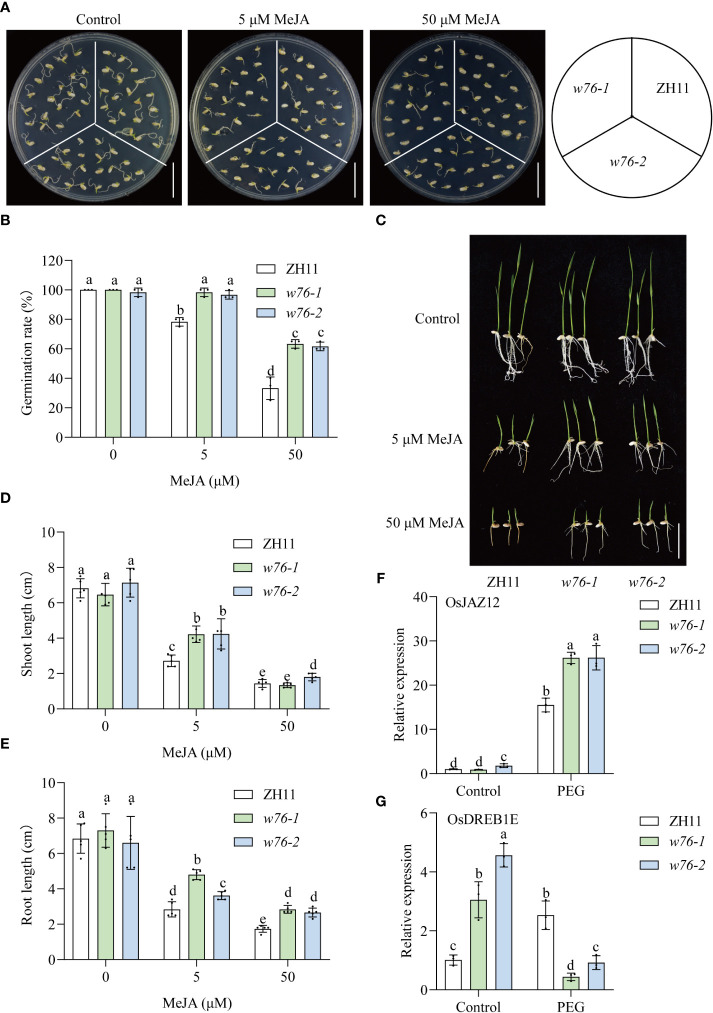
OsWRKY76 positively regulates JA signaling. **(A, B)** Germination phenotypes **(A)** and seed germination rates **(B)** of wild-type ZH11 and *OsWRKY76*-knockout mutant (*w76*-*1* and *w76*-*2*) seeds treated with MeJA for 3 days. ZH11, *w76*-*1*, and *w76*-*2* seeds were germinated on 1/2 MS medium containing 0, 5, or 50 μM MeJA. Scale bars, 5 cm. **(C–E)** Seedling phenotypes **(C)**, shoot length **(D)**, and root length **(E)** of wild-type ZH11 and *OsWRKY76*-knockout mutant (*w76*-*1* and *w76*-*2*) plants treated with MeJA for 5 days. One-day-old seedlings were transferred to 1/2 MS medium containing 0, 5, or 50 μM MeJA. Scale bars, 5 cm. **(F, G)** Reverse transcription quantitative PCR (RT-qPCR) analysis showing the expression levels of *OsJAZ12* **(F)** and *OsDREB1E* **(G)** in the *OsWRKY76*-knockout mutants (*w76*-*1* and *w76*-*2*) treated with or without 20% PEG 6000 for 3 h (n = 3; *P* < 0.05; one-way ANOVA).

Given that *OsWRKY76* expression levels were induced by MeJA treatment, we hypothesized that *OsWRKY76* is involved in the JA signaling pathway. To test whether *OsWRKY76* knockout affected sensitivity to MeJA, we performed the effects of MeJA on seed germination or seedling development among the wild-type ZH11 plants and the *OsWRKY76* mutants. As shown in **Figures 3A, B**, *OsWRKY76*-knockout mutants (*w76*-*1* and *w76*-*2*) showed hyposensitivity to exogenous MeJA compared to wild-type ZH11 during seed germination. Furthermore, MeJA treatment significantly inhibited shoot and root growth in wild-type ZH11 compared with *OsWRKY76*-knockout mutants (*w76*-*1* and *w76*-*2*) (**Figures 3C–E**). These results suggest that the loss of the OsWRKY76 function decreases MeJA sensitivity.

To further confirm this speculation, we performed RT-qPCR to examine the expression of JA signaling and drought-responsive genes under drought stress in *OsWRKY76*-knockout mutants. The expression of *OsJAZ12* (related to JA signaling) was significantly induced at 3 h of exposure to 20% PEG treatment, showing a 15-fold increase over that in wild-type ZH11 (**Figure 3F**). *OsJAZ12* expression was notably higher in *OsWRKY76*-knockout mutants (*w76*-*1* and *w76*-*2*) than in wild-type ZH11 under PEG treatment (**Figure 3F**). OsDREB1E positively regulates tolerance to drought stress (Chen et al., 2008). *OsDREB1E* expression was lower in *OsWRKY76*-knockout mutants (*w76*-*1* and *w76*-*2*) than in wild-type ZH11 subjected to drought stress, but it was upregulated under normal growth conditions (**Figure 3G**). These results indicate that the JA signaling pathway is attenuated in the *OsWRKY76*-knockout mutants under drought stress. The expression level of the drought-tolerance gene *OsDREB1E* was also repressed under drought stress. Taken together, these results suggest that OsWRKY76 positively regulates JA signaling under drought stress.

### OsWRKY76 interacts with OsJAZ12

**Figure 4:**
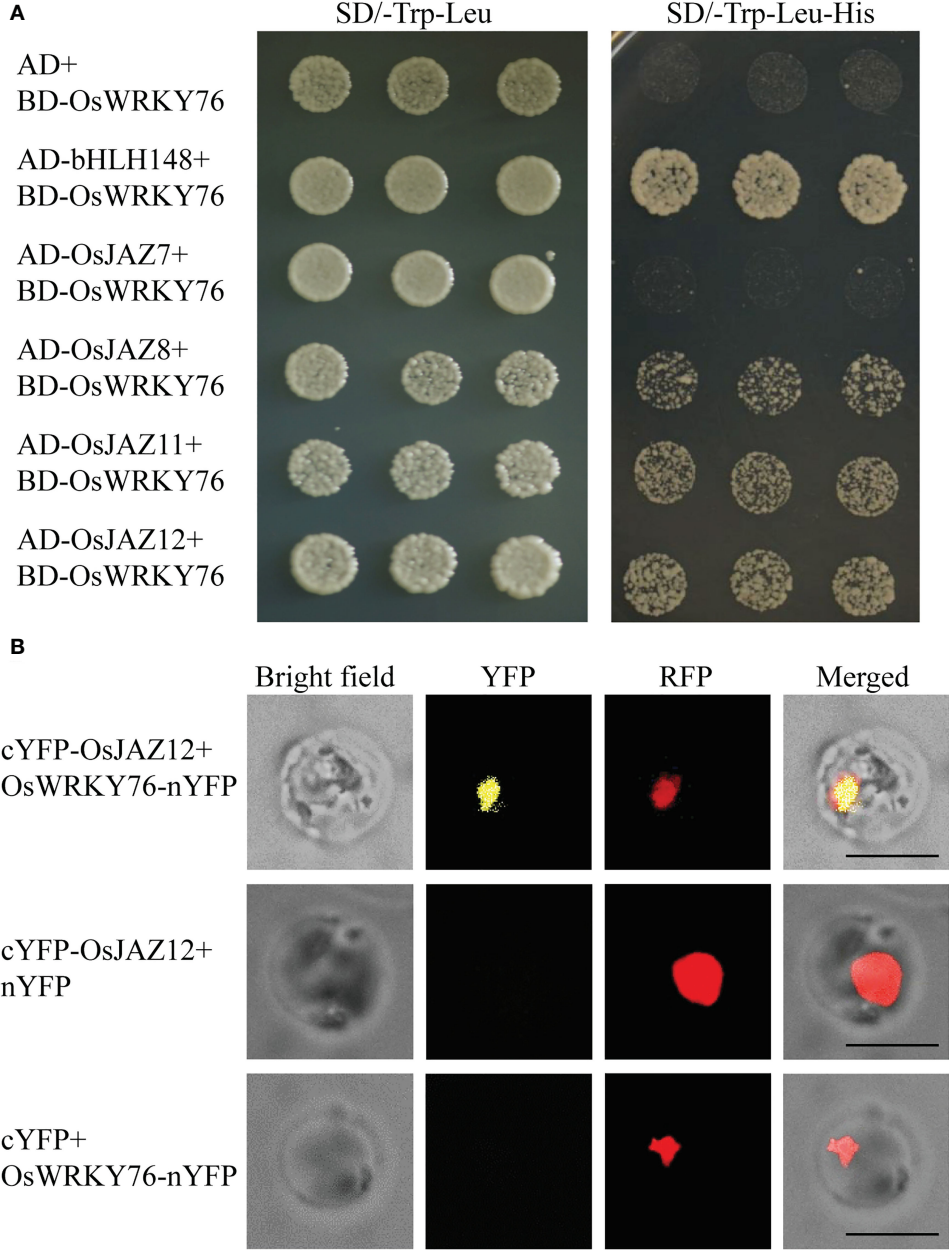
OsWRKY76 interacts with OsJAZ12. **(A)** Yeast two-hybrid assay. The pbridge-OsWRKY76 (BD-W76) bait vector was co-transformed with pGADT7 (AD), AD-OsbHLH148, AD-OsJAZ7, AD-OsJAZ8, AD-OsJAZ11, or AD-OsJAZ12 prey vector into the yeast strain Y2HGold. The positive transformants were grown on SD/-Trp-Leu and SD/-Trp-Leu-His dropout plates to identify protein–protein interactions. **(B)** Bimolecular fluorescence complementation (BiFC) assay of OsWRKY76 and OsJAZ12 interactions. cYFP or cYFP-OsJAZ12 and nYFP or OsWRKY76-nYFP were transformed into *Arabidopsis* protoplasts. Scale bars, 20 μm.

OsbHLH148 interacted with some OsJAZ proteins. The interaction between OsbHLH148 and OsJAZ12 was relatively strong, and the interaction with OsJAZ7, 8, and 11 was relatively weak (Seo et al., 2011). Our previous data showed that OsWRKY76 interacts with OsbHLH148 *in vitro* and *in vivo* (Zhang et al., 2022). To investigate whether OsWRKY76 interacted with these OsJAZ proteins, we performed a yeast two-hybrid assay. In yeast cells, OsWRKY76 interacted with OsJAZ8, 11, and 12 but not with OsJAZ7 (**Figure 4A**). The interaction between OsWRKY76 and OsJAZ12 was further confirmed using a BiFC assay in rice protoplasts (**Figure 4B**). These results indicate that OsWRKY76, OsbHLH148, and OsJAZ12 form a heterotrimer.

### OsWRKY76 competes with OsJAZ12 to bind OsbHLH148

**Figure 5:**
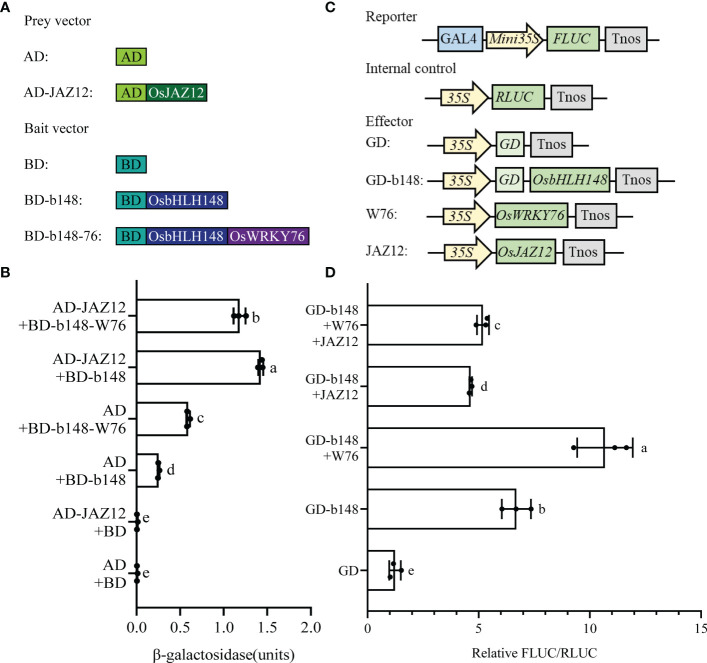
OsWRKY76 competes with OsJAZ12 to bind OsbHLH148. **(A)** In the yeast three-hybrid assay, prey vector (AD or AD-JAZ12) and bait vector (BD, BD-b148, or BD-b148-W76) construction. **(B)** Yeast three-hybrid assay. Yeast strain Y190 cells were co-transformed with a prey vector (AD or AD-JAZ12) and bait vector (BD, BD-b148, or BD-b148-W76). The positive transformants were grown on SD/-Trp-Leu-Met dropout plates, and β-galactosidase activity levels were detected. **(C)** In the Gal4-dependent chimeric transactivation assay, vector construction of the OsbHLH148 transcriptional activity assay. **(D)** Relative FLUC/RLUC of the OsbHLH148 transcriptional activity assay in rice protoplasts by co-transformation of different effector vectors (OsWRKY76 and OsJAZ12 proteins) using the Gal4-dependent chimeric transactivation assay (n = 3; *P* < 0.05; one-way ANOVA).

To test the effect of OsWRKY76 on the interaction between OsbHLH148 and OsJAZ12, we performed a yeast three-hybrid assay. When co-transformed with pGADT7 (AD) and pbridge-OsbHLH148 (BD-b148), β-galactosidase activity was significantly higher than that of the empty vector, indicating that OsbHLH148 has transcriptional activation activity in yeast cells (**Figures 5A, B**). When co-transformed with pGADT7-OsJAZ12 (AD-JAZ12) and BD-b148, β-galactosidase activity was significantly higher than when co-transformed with AD and BD-b148, and it was also significantly higher than when co-transformed with AD-JAZ12 and pbridge-OsbHLH148-OsWRKY76 (BD-b148-W76), indicating that OsWRKY76 weakens the interaction between OsbHLH148 and OsJAZ12 in yeast cells (**Figures 5A, B**).

To analyze the effect of OsWRKY76 and OsJAZ12 proteins on OsbHLH148 transcriptional activity, we performed a Gal4-dependent chimeric transactivation assay. The full-length CDS of *OsbHLH148* was amplified and fused into the GD, creating the GD-b148 effector vector (**Figure 5C**). The Gal4-dependent chimeric transactivation assay showed that the FLUC activity of the GD-b148 vector was 6-fold higher than that of the GD vector, indicating that OsbHLH148 is a transcriptional activator in rice protoplasts (**Figure 5D**). The OsWRKY76 protein activated the transactivation activity of OsbHLH148. However, the OsJAZ12 protein repressed the transactivation activity of OsbHLH148, and this repression was partly restored by OsWRKY76 (**Figure 5D**). Taken together, these results indicate that OsWRKY76 activates OsbHLH148 transcriptional activity by interfering with the association of OsJAZ12 with OsbHLH148.

### OsWRKY76 directly activates *OsDREB1E* expression in response to drought stress

**Figure 6:**
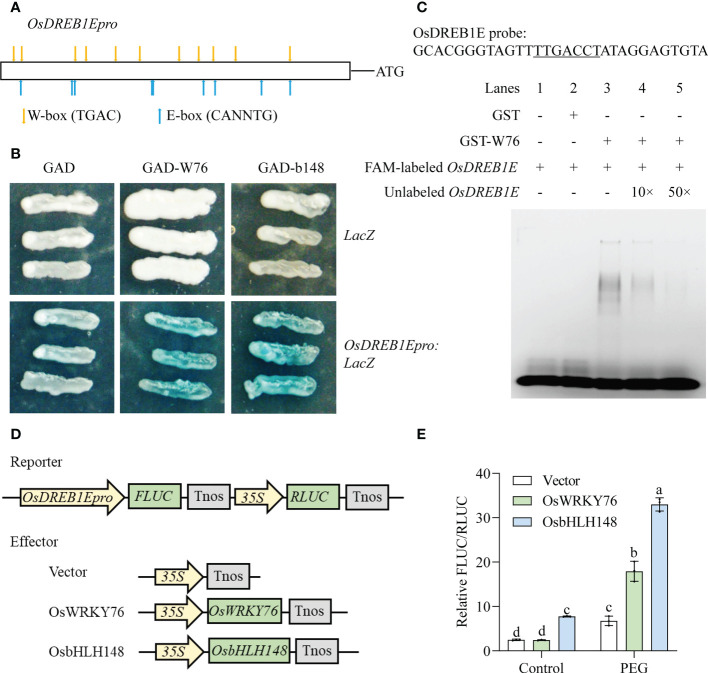
OsWRKY76 and OsbHLH148 directly regulate *OsDREB1E* expression. **(A)** Schematic of the *OsDREB1E* promoter showing the W-box and E-box elements. **(B)** Yeast one-hybrid assay. The GAL4-Activation Domain (GAD), OsWRKY76 fused with GAD (GAD-W76), or OsbHLH148 fused with the GAD (GAD-b148) constructs were separately co-transformed with the *LacZ* reporter gene driven by the *OsDREB1E* promoter (*OsDREB1Epro :* LacZ) into the yeast strain EGY48. The positive transformants were identified using SD/-Trp-Ura dropout plates containing X-Gluc for blue color development. **(C)** Oligonucleotides of the probe of the *OsDREB1E* promoter (*OsDREB1E* probe) used in the electrophoretic mobility shift assay (EMSA). The W-box elements are labeled with an underline. EMSA binding of OsWRKY76 with the *OsDREB1E* promoter containing the W-box element. The 6-FAM 5’ end-labeled *OsDREB1E* probe is shown in lanes 1–5. The 6-FAM 5’ end-labeled *OsDREB1E* probe was incubated with the glutathione S-transferase (GST)-OsWRKY76 protein (lane 3) and GST protein (lane 2), and the *OsDREB1E* probe alone (lane 1) served as the negative control. Unlabeled *OsDREB1E* probe competitors were used in 10 or 50 molar excesses (lanes 4 and 5). **(D)** Vector construction of the *OsDREB1E* promoter activity assay. The *OsDREB1E* promoter was cloned into the double vector pGreenII 0800-LUC as the reporter vector. The full-length CDSs of *OsWRKY76* and *OsbHLH148* were cloned into pCAMBIA1300 to serve as the effector vector. **(E)** OsWRKY76 and OsbHLH148 activated *OsDREB1E* promoter activity using a dual-luciferase assay (relative FLUC/RLUC) (n = 3; *P* < 0.05; one-way ANOVA).

*OsDREB1E* was activated in *OsWRKY76*-knockout mutants (*w76*-*1* and *w76*-*2*) under normal conditions, but the activity showed repression under drought stress (**Figure 3G**). There were several W-box elements (TTGACT/C, WRKY recognition sites) and E-box elements (CANNTG, bHLH recognition sites) in the *OsDREB1E* promoter region using the PlantCARE database (**Figure 6A**). Therefore, we investigated whether OsWRKY76 and OsbHLH148 bound to the *OsDREB1E* promoter to confer drought tolerance in rice. We performed a yeast one-hybrid assay, which revealed direct binding of OsWRKY76 and OsbHLH148 to the *OsDREB1E* promoter (**Figure 6B**). To further verify that OsWRKY76 bound to the *OsDREB1E* promoter, we performed an EMSA. Strong mobility shift bands in the *OsDREB1E* probe containing the W-box element (**Figure 6C**) were detected for the GST-OsWRKY76 protein (lane 3) but not for the GST protein (lane 2) or the *OsDREB1E* probe alone (lane 1) (**Figure 6C**). Strong mobility shift bands were reduced when increasing amounts (10× and 50×) of the unlabeled *OsDREB1E* competitor probe were added (lanes 3–5) (**Figure 6C**). Thus, EMSA showed that OsWRKY76 bound directly to the *OsDREB1E* promoter containing the W-box element *in vitro*.

To investigate whether OsWRKY76 and OsbHLH148 regulated the transcription of *OsDREB1E*, we conducted dual-luciferase assays in rice protoplasts. The *OsDREB1E* promoter was fused with the *FLUC* reporter gene, and *OsWRKY76* or *OsbHLH148*, driven by 35S, was the effector (**Figure 6D**). There was no significant difference in the relative FLUC/RLUC activity of co-transfection with the *35S:OsWRKY76* vector and with the empty vector under normal conditions (**Figure 6E**). When the transformed protoplasts were treated with PEG treatment for 20 min, the transcription of *OsDREB1E* was strongly activated by OsWRKY76 (**Figure 6E**). Under normal conditions and PEG treatment, the relative FLUC/RLUC activity co-transfected with *35S:OsbHLH148* was significantly higher than that of the empty vector, indicating that OsbHLH148 elevated *OsDREB1E* promoter activity in rice protoplasts (**Figure 6E**). These results revealed that OsWRKY76 and OsbHLH148 directly activated the transcription of *OsDREB1E* under drought stress.

## Discussion

As global climate change continues to cause water scarcity, drought is emerging as a prevalent and important stress factor for crop production worldwide. The latest FAO data have shown that drought has cost the world $29 billion in crop losses over the past decade (https://www.fao.org/, accessed 24 May 2022). Rice serves as a staple crop for more than half of the world’s population (Seck et al., 2012). The identification of drought-tolerance genes can speed up the development of drought-tolerant rice varieties. A few WRKY TFs conferring drought tolerance in rice, including OsWRKY11, OsWRKY30, OsWRKY47, and OsWRKY87, have been identified (Shen et al., 2012; Raineri et al., 2015; Lee et al., 2018; Yan et al., 2021). In this study, *OsWRKY76* was regulated by drought stress, and its knockout weakened drought tolerance at the seedling stage (**Figures 1** and **2**).

Under drought stress, plants regulate adaptive and physiological responses through the metabolism of plant hormones and their signaling pathways. JA is a key phytohormone that regulates plant drought stress adaptation. In pepper (*Capsicum annuum* L.), CaCIPK3 increases drought and MeJA tolerance by enhancing the expression of JA-related genes (Ma et al., 2021). In *Arabidopsis*, JASMONIC ACID OXIDASES (JAO1–4) catalyze the specific oxidation of JA to 12OH-JA for attenuating JA-Ile formation and the JA signaling pathway (Smirnova et al., 2017). *JAO2* mutants possess higher JA-Ile signaling and drought survival compared with wild-type plants, which is further enhanced by JAO3 and JAO4 deficiency (Marquis et al., 2022). *AtNOG1*-*1* or *AtNOG1*-*2* overexpression in rice positively regulates the expression of genes related to the JA signaling pathway and stomata regulation to prevent water loss (Pant et al., 2022). In sea buckthorn (*Hippophae rhamnoides* L.), HrTCP20 confers drought tolerance by mediating the JA signaling pathway (Yao et al., 2022). MeSPL9 weakens drought tolerance by regulating protectant metabolite contents and JA signaling in *cassava* (Li et al., 2022). In this study, *OsWRKY76* knockout decreased sensitivity to exogenous MeJA in seed germination and seedling development (**Figures 3A–E**). JAZ proteins suppress various TF activities and negatively regulate the JA signaling pathway (Ke et al., 2015). Our research showed that *OsJAZ12* was significantly induced by drought stress, and its expression was significantly higher in *OsWRKY76*-knockout mutants than in wild-type ZH11 under drought stress (**Figure 3F**). And then, JAZ12 protein dramatically suppressed activities of various TF participating in the JA signaling pathway. Based on these findings, we suggest that OsWRKY76 positively regulates drought stress in a JA-dependent signaling pathway.

Under normal conditions, JAZ proteins interacted with bHLH148 and suppressed its activity, causing an inhibition state of JA signaling in rice (Seo et al., 2011; Ke et al., 2015). When rice plants suffer from drought stress, JA levels are elevated, and JA promotes the interaction of JAZ proteins with COI1 of the SCF^COI1^ complex and leads to JAZ protein degradation, which releases bHLH148 and activates bHLH148 activity (Mosblech et al., 2011; Seo et al., 2011; Wasternack and Song, 2017). Our data showed that OsWRKY76 interacted with OsJAZ proteins and weakened the interaction between OsbHLH148 and OsJAZ12 in yeast cells (**Figures 4** and **5B**). The OsJAZ12 protein repressed the transactivation activity of OsbHLH148, and this repression was partly restored by OsWRKY76 (**Figure 5D**). *OsJAZ12* expression was notably higher in *OsWRKY76*-knockout mutants than in wild-type ZH11 under drought treatment (**Figure 3F**). Based on the results, we propose that OsWRKY76 plays an essential role in OsbHLH148-mediated JA signaling. However, OsWRKY76 is upstream of OsbHLH148 in drought stress, lacking genetic evidence for the double mutants of OsWRKY76 and OsbHLH148. In future work, we should analyze the double mutants of OsWRKY76 and OsbHLH148 phenotypes of drought stress by compared with single gene mutants, which could further help to understand their relationships.

Induced stress responses allow plants to survive under a wider range of environmental conditions (VanWallendael et al., 2019). The extreme environment triggers overall and rapid reprogramming events in cells (Wu et al., 2021). Transcription of most *DREB* genes in various plant species exhibits relatively low basal expression levels but is rapidly induced by different environmental stressors (Agarwal et al., 2017). In rice, *OsDREB1E* could be significantly induced and positively regulate tolerance to drought stress (Chen et al., 2008). Here, *OsDREB1E* expression was lower in *OsWRKY76*-knockout mutants than in wild-type ZH11 under drought stress, but it was upregulated under normal growth conditions (**Figure 4G**). OsWRKY76 bound directly to the *OsDREB1E* promoter and activated *OsDREB1E* expression in response to drought stress (**Figure 6**). Similarly, SEUSS is transcriptional co-repressors that downregulates gene expression during flower development in *Arabidopsis thaliana* (Grigorova et al., 2011). Interestingly, SEUSS promotes the expression of auxin-related genes and positively regulates warm temperature-mediated hypocotyl growth (Huai et al., 2018). Moreover, OsMADS57 could suppress *OsWRKY94* activity under normal temperatures but activates its transcription in response to cold stress (Chen et al., 2018). Therefore, OsWRKY76 activated *OsDREB1E* transcription under drought stress instead of under normal growth conditions, which avoids incurring costly fitness trade-offs and may act as a molecular link between drought tolerance and plant growth. Moreover, OsWRKY76 responds to multiple biotic and abiotic stresses (Yokotani et al., 2013). OsWRKY76 positively regulates cold stress by enhancing *OsDREB1B* transcription (Zhang et al., 2022). Thus, OsWRKY76 is not only a positive regulator of cold tolerance in rice, but also it confers the drought tolerance of rice at the seedling stage. OsWRKY76 may be involved in multiple resistance of rice, but their mechanisms may be different.

## Conclusions

**Figure 7:**
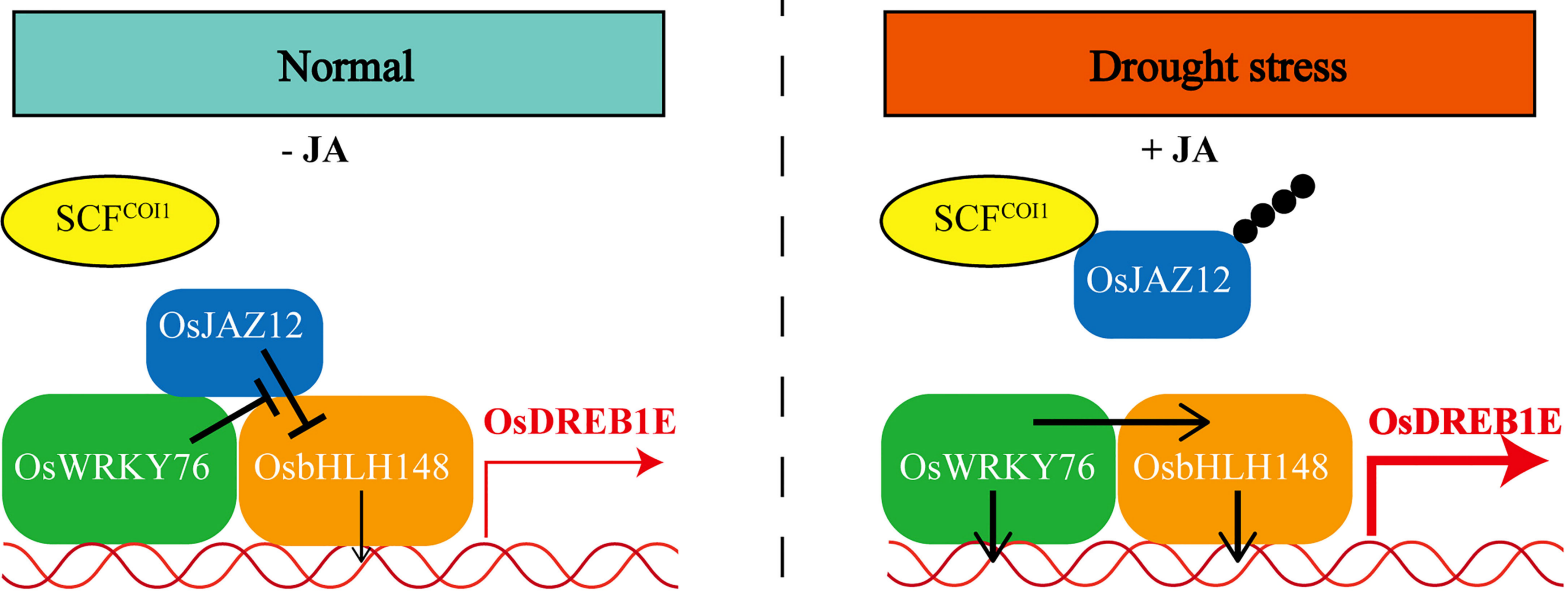
A model illustrating that OsWRKY76 positively regulates drought stress by OsbHLH148-mediated jasmonate signaling in rice.

In the present study, our findings support a model in which OsWRKY76 acts as a key regulator in drought stress and jasmonate signaling in rice (**Figure 7**). Under normal growth conditions, OsWRKY76 interacted with OsJAZ proteins and weakened the interaction between OsbHLH148 and OsJAZ12. Furthermore, the OsJAZ12 protein repressed the transactivation activity of OsbHLH148, and this repression was partly restored by OsWRKY76. Under drought stress, JA-Ile levels were elevated, leading to the degradation of OsJAZ12 and the activation of *OsWRKY76* and *OsbHLH148* expressions. OsWRKY76 and OsbHLH148 directly activated the transcription of *OsDREB1E* and positively regulated drought tolerance.

## Data availability statement

The original contributions presented in the study are included in the article/**Supplementary Material**. Further inquiries can be directed to the corresponding authors.

## Author contributions

TW, XD and MZ designed the study. MZ performed experiments, data analysis, and wrote the manuscript. RZ, KH, ZW and BG assisted in performing part of the experiments. KH, SH, ZL and WJ assisted in analyzing the data XD and TW supervised the project and modified the manuscript. All authors contributed to the article and approved the submitted version.
