# Supplementary material for: OsWRKY76 positively regulates drought stress via OsbHLH148-mediated jasmonate signaling in rice
Source: Front Plant Sci. 2023 Apr 5;14:1168723. doi: 10.3389/fpls.2023.1168723 (PMC10113545; doi:10.3389/fpls.2023.1168723)
Supplement: Supplementary file 1 [file DataSheet_1.docx]

Supplementary Material


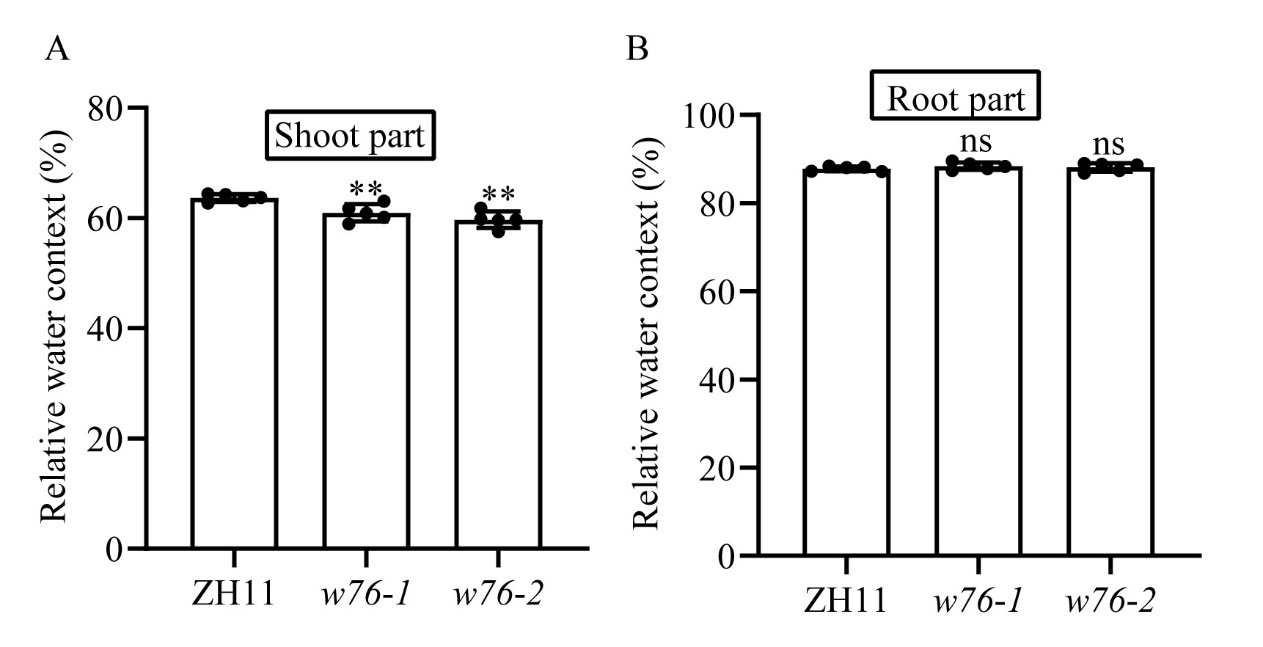


**Supplementary Figure 1.** OsWRKY76 positively regulates rice drought stress tolerance.

(**A,B**) Relative water content of shoot (**A**) and root (**B**) parts of ZH11 and *OsWRKY76*-knockout mutants (*w76*-*1* and *w76*-*2*) rice seedlings under 10 days of 20% PEG treatment.

**Supplementary Table 1.** List of primers used in this study.

| Primer name | Forward sequence (5'-3') | Reverse sequence (5'-3') |
| --- | --- | --- |
| (a) Primers for RT-qPCR analysis | | |
| OsWRKY76, Yokotani *et al.* (2013) | TTCCGAATGCTTTTCTGCTG | ATCGTGAGGCCCGATAGAAG |
| OsDREB1E | ACTTCCCTTGCTACCCGATG | GGCTCGATGAGCATTCCCTG |
| OsJAZ12 | GACGACGGGTTCAGGTTCG | AGTAGCTCCAGAGCGGCAT |
| Ubiquitin5, Mao *et al.* (2019) | ACCACTTCGACCGCCACTACT | ACGCCTAAGCCTGCTGGTT |
| ACTIN | CCTGGCAGTATGAAGGTAGTTG | GAAGCACTTCATGTGGACGAT |
| (b) Primers for yeast two- and three-hybrid assays | | |
| AD-OsbHLH148 | GGAGGCCAGTGAATTCATGCAAATGGAGTCGTACTACG | CGAGCTCGATGGATCCTCAAAACACATTTTGCACATGA |
| AD-OsJAZ7 | GGAGGCCAGTGAATTCATGGCGGCTTCCGCGAGG | CGAGCTCGATGGATCCTCATTGGCCGCGTTCTATGGG |
| AD-OsJAZ8 | GGAGGCCAGTGAATTCATGGCCGGCCGTGCGACGGC | CGAGCTCGATGGATCCTCATATCTCCTGCTTTATTG |
| AD-OsJAZ11 | GGAGGCCAGTGAATTCATGGCCGGTAGTAGCGAGCA | CGAGCTCGATGGATCCTCACAGGCTGAGAGTGGGGTTC |
| AD-OsJAZ12 | GGAGGCCAGTGAATTCATGGCCGCCGCCGGCAGC | CGAGCTCGATGGATCCTCAGAGCCCGAGCCATGTCGCC |
| BD-OsWRKY76 | TGTATCGCCGGAATTCATGGACGCGGCGTGGCG | TTGGCTGCAGGTCGACCTAGAATTCGGGCAGCTTCTGGAGG |
| BD-OsbHLH148 | TGTATCGCCGGAATTCATGCAAATGGAGTCGTACTACG | TTGGCTGCAGGTCGACTCAAAACACATTTTGCACATGA |
| BD-OsbHLH148- OsWRKY76 | AAGAGAAAGGTGGCGGCC ATGGACGCGGCGTGGCG | CTTCGGGCTAATGCGGCCGC GAATTCGGGCAGCTTCTGGAGG |
| (c) Primers for BiFC assay | | |
| OsWRKY76-nYFP | AGGTACCCGGGGATCCATGGACGCGGCGTGGCGCGG | CGCCGTCGACTCTAGAGAATTCGGGCAGCTTCTGGA |
| cYFP-OsJAZ12 | CAAGGCCGGCGGATCCATGGCCGCCGCCGGCAGC | GCAGGTCGACTCTAGATCAGAGCCCGAGCCATGTCGCC |
| (d) Primers for Gal4-dependent chimeric transactivation assay | | |
| 35S:GD-OsbHLH148 | TAGAACTAGTGGATCCATGCAAATGGAGTCGTACTACG | GCTTGATATCGAATTCTCAAAACACATTTTGCACATGA |
| 35S:OsWRKY76 | CGAGTGGCCACCATGGGCGAGCTCATGGACGCGGCGTGGCG | GCAGCCCGGGGGATCCCTAGAATTCGGGCAGCTTCTGGAGG |
| 35S:OsJAZ12 | CGAGTGGCCACCATGGGCGAGCTCATGGCCGCCGCCGGCAGC | GCAGCCCGGGGGATCCTCAGAGCCCGAGCCATGTCGCC |
| (e) Primers for yeast one-hybrid assay | | |
| OsDREB1Epro:LacZ | TATTGGATCGGAATTCTTCTATGTCAATCATCTACT | ATGCCTCGAGGTCGACCGATGGATCAGTTCACTCGA |
| GAD-OsWRKY76 | TGCCTCTCCCGAATTCATGGACGCGGCGTGGCG | TCCAAAGCTTCTCGAGCTAGAATTCGGGCAGCTTCTGGAGG |
| GAD-OsbHLH148 | TGCCTCTCCCGAATTCATGCAAATGGAGTCGTACTACG | TCCAAAGCTTCTCGAGTCAAAACACATTTTGCACATGA |
| (f) Primers for EMSA | | |
| GST-OsWRKY76 | TCCAGGGGCCCCATATGATGGACGCGGCGTGGCG | GGAATTCTAGACATATGCTAGAATTCGGGCAGCTTCTGGAGG |
| OsDREB1Epro-probe | GCACGGGTAGTTTTGACCTATAGGAGTGTA | TACACTCCTATAGGTCAAAACTACCCGTGC |
| (g) Primers for dual-luciferase assay | | |
| OsDREB1Epro:Luc | CGGTATCGATAAGCTTTTCTATGTCAATCATCTACT | TAGAACTAGTGGATCCCGATGGATCAGTTCACTCGA |
| 1300-OsWRKY76 | ACGATGATAAGGGCGGTACC ATGGACGCGGCGTGGCG | AGGCTACGTAGGATCCCTAGAATTCGGGCAGCTTCTGGAGG |
| 1300-OsbHLH148 | ACGATGATAAGGGCGGTACC ATGCAAATGGAGTCGTACTACG | AGGCTACGTAGGATCCTCAAAACACATTTTG CACATGA |

**Supplementary Table 2** List of the accession numbers of genes used in this study.

| Gene name | Accession number |
| --- | --- |
| OsWRKY76 | LOC_Os09g25060 |
| OsbHLH148 | LOC_Os03g53020 |
| OsDREB1E | LOC_Os04g48350 |
| OsJAZ7 | LOC_Os07g42370 |
| OsJAZ8 | LOC_Os09g26780 |
| OsJAZ11 | LOC_Os03g08320 |
| OsJAZ12 | LOC_Os10g25290 |
| Ubiquitin5 | LOC_Os01g22490 |
| ACTIN | LOC_Os01g64630 |
